# Regional oximetry for diagnosing compartment syndrome: a scoping review

**DOI:** 10.1186/s13018-026-06763-x

**Published:** 2026-02-23

**Authors:** Linda Yuhan Tang, Dave Osinachukwu Duru, Andrew Browne, Andrew Kailin Zhou, Saroop Nandra, Matija Krkovic

**Affiliations:** 1 School of Clinical Medicine, University of Cambridge, Cambridge, CB2 0SP UK; 2 Nuffiled Department of Population Health, University of Oxford, Oxford, UK; 3 University of Warwick, Warwick, UK; 4 Vascular Surgery Department, University Hospital Coventry and Warwickshire, Coventry, UK; 5 Trauma and Orthopaedics Department, University Hospital Coventry and Warwickshire, Coventry, UK; 6 Department of Trauma and Orthopaedics, Addenbrookes Major Trauma Unit, Cambridge University Hospitals, Cambridge, UK

**Keywords:** Compartment syndrome, Regional oximetry, Near-infrared spectroscopy, Ischemia monitoring, Intracompartmental pressure, Non-invasive diagnostics

## Abstract

**Purpose:**

Diagnosis of compartment syndrome remains challenging, as intracompartmental pressure (ICP) monitoring measures mechanical pressure rather than tissue perfusion. Near-infrared spectroscopy (NIRS) enables non-invasive, continuous assessment of tissue oxygen saturation (StO_2_), potentially identifying ischemia earlier. However, its diagnostic accuracy remains uncertain.

**Methods:**

Following PRISMA-ScR guidelines, PubMed, EMBASE, Cochrane Library, ClinicalTrials.gov, and WHO-ICTRP were searched to April 2025 for studies evaluating NIRS in acute (ACS) or chronic exertional (CECS) compartment syndrome. Data on diagnostic accuracy, device protocols, and patient characteristics were extracted. Studies reporting comparable StO_2_ data in CECS and controls were pooled using a random-effects meta-analysis.

**Results:**

Twenty-three studies (*n* = 1000) were included. In ACS, some demonstrated strong correlation with perfusion pressure and post-fasciotomy StO_2_ recovery, while others found poor agreement with ICP or no diagnostic discrimination. There was heterogeneity in device type, patient demographics (particularly skin pigmentation), and protocols. In CECS, pooled analysis showed lower baseline StO_2_ (mean difference − 3.4%, 95% CI − 6.2 to − 0.7) and greater exercise-induced deoxygenation (+ 15.0%, 95% CI 0.4–29.7) versus controls.

**Conclusion:**

NIRS provides a physiologically relevant but technically variable indicator of compartmental perfusion, which may complement, but not replace, ICP monitoring for compartment syndrome. The results presented are hypothesis-generating and require prospective trials with standardised protocols, inclusive calibration, and prospective validation before clinical adoption of NIRS.

## Introduction

Compartment syndrome occurs when pressure within a closed fascial compartment exceeds capillary perfusion pressure, leading to ischemia and potentially irreversible tissue necrosis [1]. Acute compartment syndrome (ACS) is a surgical emergency that often follows fractures, crush injuries, or reperfusion after ischemia. Prompt diagnosis and fasciotomy are critical to prevent irreversible damage, limb loss, or death [2]. Clinically, ACS presents early with tense swelling and pain out of proportion to the injury, particularly on passive stretch. Paralysis and pulseless are late findings [3]. However, the early signs require a communicative, alert patient, complicating diagnosis in sedated, intubated, or non-verbal patients [4].

Chronic exertional compartment syndrome (CECS), is a reversible ischemic disorder triggered by repetitive exercise, commonly affecting the lower limbs of young athletes and military personnel [5]. Diagnosis relies on symptom reproduction during exertion, supported by intracompartmental pressure (ICP) testing.

ICP monitoring remains the conventional objective adjunct in both ACS and CECS. Diagnostic thresholds typically include an absolute ICP > 40 mmHg or a differential pressure (ΔP = diastolic blood pressure − ICP) < 30 mmHg [6]. However, ICP measurement is invasive, painful, and prone to variability in technique and threshold definition [7]. Although sensitive, ICP is not specific, as pressures may be elevated due to edema or swelling even when perfusion is adequate, occasionally leading to unnecessary fasciotomy [2].

Regional oximetry using near-infrared spectroscopy (NIRS) offers a non-invasive method for assessing tissue oxygenation within the affected compartment. Based on the Beer–Lambert law, NIRS emits near-infrared light into tissue and quantifies oxygenated versus deoxygenated haemoglobin to calculate tissue oxygen saturation (StO_2_) [8]. Because both ACS and CECS involve compromised oxygen delivery, NIRS provides a direct physiological indicator of perfusion rather than a mechanical surrogate of pressure [9]. Crucially, NIRS can be used in sedated or non-verbal patients or in paediatric settings and provides continuous monitoring, features that enhance its potential role as an adjunct or early warning system [4]. Over the past two decades, studies in animal models, healthy volunteers, and patients with acute or chronic compartment syndromes have explored NIRS for diagnosis, monitoring, and postoperative evaluation [2, 9].

This scoping review systematically maps the available evidence on the use of NIRS-based regional oximetry for diagnosing acute and chronic compartment syndrome. It compares diagnostic performance with ICP monitoring, highlights its utility in non-verbal patients and paediatric settings, and identifies current methodological gaps to guide future clinical research and potential guideline integration.

## Methods

This scoping review was conducted to systematically evaluate the incidence, mechanisms, and outcomes of “Regional Oximetry in Diagnosing Compartment Syndrome.” The methodology adhered to the PRISMA-ScR (Preferred Reporting Items for Systematic Reviews and Meta-Analyses Extension for Scoping Reviews) guidelines, which provide a structured framework for exploring broad research questions and identifying gaps in the literature [10].

### Inclusion and exclusion criteria

We included any study or report (clinical trials, observational studies, case series, case reports, and experimental laboratory studies) that evaluated the use of regional oximetry (e.g. near-infrared spectroscopy-based tissue oxygen saturation monitoring) for diagnosing or detecting compartment syndrome in any anatomic location. Both acute compartment syndrome (typically post-traumatic or postoperative) and chronic exertional compartment syndrome were included. No age restrictions were imposed. Excluded studies included conference abstracts, pre-clinical studies (e.g. animal models or cadaveric studies), and publications not in English.

A thorough search was conducted in the Cochrane Library, PubMed, EMBASE and ClinicalTrials.gov and World Health Organization International Clinical Trials Registry Platform (WHO-ICTRP) from their inception to April 30, 2025. To identify relevant literature, keywords included: “compartment syndrome,” “acute compartment syndrome,” “chronic exertional compartment syndrome,” “compartment pressure,” “near-infrared spectroscopy,” “tissue oximetry,” and “regional oxygen saturation.” The search for PubMed was: (((acute compartment syndrome) OR (compartment pressure)) OR (“Compartment Syndromes”[Mesh] OR “Chronic Exertional Compartment Syndrome”[Mesh])) AND ((((near-infrared spectroscopy) OR (tissue oximetry)) OR (regional oxygen saturation)) OR ((“Oxygen Saturation”[Mesh]) OR (“Oximetry”[Mesh] OR “Blood Gas Monitoring, Transcutaneous”[Mesh]))). Reference lists of retrieved articles and prior reviews were also screened for additional sources.

**Fig. 1:**
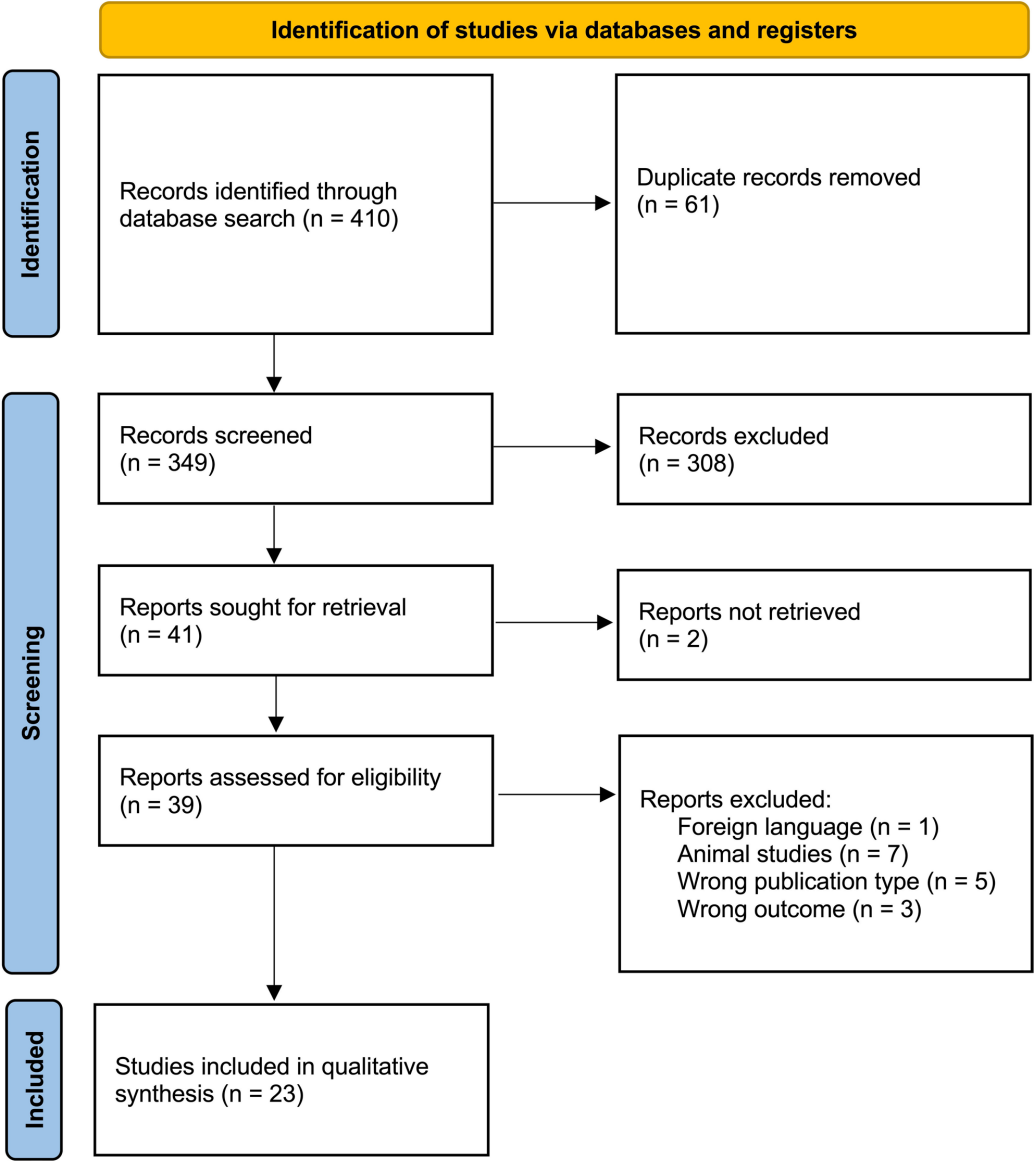
PRISMA flow diagram of studies

Two independent reviewers screened titles and abstracts of retrieved articles against the inclusion and exclusion criteria. Full texts were then evaluated to confirm relevance. Discrepancies in study inclusion were resolved through consensus or consultation with a third reviewer to minimise bias. A PRISMA flow diagram summarising the study selection process was prepared (Fig. 1).

Key data extracted included study design, population characteristics (e.g. age, ethnicity, sex), details of the oximetry method (device used, placement of sensors), reference standard for compartment syndrome diagnosis (e.g. clinical criteria, intracompartmental pressure measurements, surgical findings), and main outcomes regarding diagnostic effectiveness (such as sensitivity/specificity of near-infrared spectroscopy, oxygenation thresholds identified, time course of changes, etc.).

Given the scoping nature of the review, formal quality appraisal of studies was not undertaken; instead, results are summarised broadly to map the state of evidence. We further noted differences between acute and chronic compartment syndrome contexts. We also specifically noted any findings or commentary on use in sedated/unresponsive patients.

### Exploratory quantitative synthesis

Quantitative data that were directly comparable across studies were identified and extracted for pooled analysis. Eligible studies reported mean and standard deviation (SD) values for near-infrared spectroscopy (NIRS)-derived tissue oxygen saturation (StO_2_) in both chronic exertional compartment syndrome (CECS) and asymptomatic control groups. Formal subgroup meta-analysis between ACS and CECS populations was not feasible due to limitations and heterogeneity within the data. Three parameters were selected: (1) relative change in StO_2_ (percentage decline from baseline to peak exercise), (2) absolute change in StO_2_ (percentage-point difference between baseline and minimum values), and (3) baseline (resting) StO_2_ prior to exercise, based on having at least three studies that measured the same outcome within a comparably defined CS and control group. Meta-analyses were conducted using R (v4.5.1, ‘metafor’ package). For continuous variables, mean differences (MDs) with corresponding 95% confidence intervals (CIs) were calculated. Studies were weighted using the inverse-variance method, and pooled effect sizes were generated under both common-effect (fixed) and random-effects models to account for between-study variability. Statistical heterogeneity was assessed using Cochran’s Q test [11] and Higgins and Thompson’s I^2^ statistic [12], with > 75% interpreted as high heterogeneity [13]. Given the small number of studies, the random-effects model was used preferentially to provide a more conservative estimate of variance. A 95% CI crossing zero was considered statistically non-significant. Significance was set at *p* < 0.05.

## Results

### Search results

A total of 410 studies were identified in the original search. After de-duplication, 349 studies were identified for title and abstract screening and 308 were excluded, leaving 41 studies for full-text screening. Based on the inclusion and exclusion criteria, 23 studies were included for final analysis (Table 1). Of these, four were included for a meta-analysis. Publication dates ranged from 1997 to 2023. The mean and median year of publication was 2011. The Preferred Reporting Items for Systematic Reviews and Meta-Analyses (PRISMA) flow diagram is indicated in Fig. 1.

**Table 1:** Characteristics of included studies investigating regional oximetry in compartment syndrome

Author	Year	Study type	Sample size (M/F)	ACS/CECS	Ethnicity	Location of NIRS probe	Timing of monitoring (pre/intra/post op), rest/exercise	How increased ICP was induced	NIRS sensor
Mohler et al.	1997	Case–control study	28	CECS	–	Over the tibialis anterior muscle	Before, during, and ten minutes after exercise	Isokinetic exercise protocol	Run-Man NIRS spectrometer
Breit et al.	1997	Cross-sectional study	10 (7 M/3F)	CECS	–	Over the tibialis anterior muscle	During inflation of the cuff, 14-min of cyclic isokinetic dorsiflexion and plantar flexion of the ankle	Applying external compression through a wide inflatable cuff	Run-Man NIRS spectrometer
Giannotti et al.	2000	Case–control study	42	ACS	–	All four leg compartments	Pre- and post-fasciotomy	Trauma and injury	Biospectrometer-NB Oximeter
Gentilello et al.	2001	Experimental trial	15 (7 M/8F)	ACS	–	Over the tibialis anterior muscle	At 30-min intervals while compression was increased	Induced calf compression model	Biospectrometer-NB Oximeter
van den Brand et al.	2004	Prospective cohort study	21	CECS	Caucasian	Over the tibialis anterior muscle	Every 3.5 s throughout the entire protocol	Exercise protocol	InSpectra tissue spectrometer
van den Brand et al.	2005	Prospective cohort study	50 (42 M/8F)	CECS	Not explicitly reported. But one patient's NIRS data could not be registered due to a dark skin tone	Over the tibialis anterior muscle	Pre- and post-fasciotomy	Clinical suspicion of CECS	InSpectra tissue spectrometer
Tobias and Hoernschemeyer	2007	Case report	1	ACS	–		Pre- and post-fasciotomy	Post-surgery	INVOS oximeter
Arató et al.	2009	Cross-sectional study	16 (12 M/4F)	ACS	–		Second post-op day	Surgery	InSpectra tissue spectrometer
Shuler et al.	2010	Prospective cohort study	14 (14 M/0F)	ACS	8 Black, 4 White, 1 Hispanic, 1 Asian	All four leg compartments	Within one hour after the clinical diagnosis of the compartment syndrome had been determined clinically	Lower extremity trauma	INVOS oximeter
Shuler et al.	2011	Case series	3 (3 M/0F)	ACS	Caucasian, Hispanic, Asian		Pre- and post-op	Trauma	INVOS oximeter
Sanchez de Toledo et al.	2011	Case report	1 (1 M/0F)	ACS	White			Post-surgery	INVOS oximeter
Bariteau et al.	2011	prospective cohort study	7 (6 M/1F)	ACS	–	All four leg compartments	Pre-op	Trauma	InSpectra tissue spectrometer
Zhang et al.	2012	Cross-sectional study	176 (73 M/103F)	CECS	–	Over the tibialis anterior muscle	Before, during, and after exercise	Exercise protocol	Run-Man NIRS spectrometer
Lee et al.	2013	Experimental study (simulation model)	15	ACS	–	Over the tibialis anterior muscle	Before and during the protocol	External compression in a chamber	INVOS oximeter
Reisman et al.	2013	Experimental study	20 (14 M/6F)	ACS	14 White, 3 Black, 2 Asian, 1 Hispanic	Over the tibialis anterior muscle	Before and during the protocol	Tourniquet	INVOS oximeter
Rennerfelt et al.	2016	Cross-sectional study	159 (76 M/83F)	CECS	–	Over the tibialis anterior muscle	Before, during, and after exercise	Induced by exercise	InSpectra tissue spectrometer
Challa et al.	2017	Experimental study	8 (5 M/3F)	ACS	–	Over the tibialis anterior muscle	During the protocol	An external pneumatic leg pressure chamber	CareGuideNIRS-pH
Gustafsson et al.	2017	Experimental study	40 (19 M/21F)	CECS	–	Over the tibialis anterior muscle	Before, during, and after the protocol	Thigh arterial cuff occlusion and treadmill running	InSpectra tissue spectrometer
Shuler et al.	2018	prospective Cohort study	109 (88 M/21 F)	ACS	Black, White, Hispanic	All four leg compartments	Up to 48 h	High-energy trauma	Equanox oximeter
Schmidt et al.	2018	Prospective cohort study	185	ACS	light, medium, and dark pigmentation	Over the tibialis anterior muscle	Between 24 and 72 h of data collection	Lower-leg injuries	Equanox oximeter
Aedo-Martín et al.	2019	Case series	2 (1 M/1F)	ACS	–		Before and after surgery	Trauma and spontaneous occuring	INVOS oximeter
Jagadeesan et al.	2022	Cross-sectional study	30	ACS	–	All four leg compartments	For three days from admission	Trauma	A custom device developed at K.J. Hospital
Tønning et al.	2023	Case–control study	48 (28 M/20F)	CECS	–	All four leg compartments	Before, during, and after the protocol	Treadmill exercise protocol	Portamon

Of the included studies, six were prospective cohort studies, five were cross-sectional studies, two were case series, two were case reports, three were case–control studies, and five were experimental studies involving human subjects.

### Patient demographics

A total of 1000 patients were included. Of these, 303 had confirmed diagnosis of compartment syndrome. In studies involving such cases, the rest of the patient population served as controls (either non-compartment syndrome related pain or healthy controls), often age or sex matched [14, 15]. Some studies included groups at risk of compartment syndrome but without confirmed diagnosis [16, 17]. When reported, compartment syndrome typically presented unilaterally. One study reported exclusively bilateral cases [18], while another described a minority of bilateral cases [14]. In studies including single series of unilateral compartment syndrome patients, a non-affected limb was used as an internal control. Fifteen studies focused on acute compartment syndrome (ACS) while eight studies investigated chronic exertional compartment syndrome (CECS). One study investigated chronic forms in type 1 diabetic patients [19]. Some studies simulated ACS using healthy human volunteers [20–23] either with prolonged application of a tourniquet or pressure cuff around the limb. One study simulated CECS using healthy volunteers [24].

Most investigated patients were male (58%) and adults. Mean age in most included studies ranged from 23 to 43 years. Of the CECS patients, a significant portion were in the military and young. Conversely, the single case studies of ACS or CECS differed in their patient populations. There were two pediatric cases of ACS: single case in a 1-month-old with Trisomy 21 [25], and a single 15-year-old male [26]. One case series involved two patients, aged 45 and 87 [27].

There was marked heterogeneity in the ethnic composition of the included studies. Ethnicity or race was reported in seven studies, within which 41% of participants were Black, 51% were White, 4% were Hispanic, and 2% were Asian. One additional study reported only skin pigmentation rather than race, describing 60% of patients as having light pigmentation, 16% as medium, and 16% as dark, with 14% unrecorded [17]. Three studies excluded Black or “dark-skinned” participants due to known inaccuracies of regional oximetry in individuals with highly melanised skin [15, 18, 28]. Across studies, the influence of skin pigmentation on regional oximetry accuracy was not reported consistently.

Studies were categorised based on patient alertness. Most involved alert participants, typically patients with chronic exertional compartment syndrome who could report pain or discomfort during exercise [15, 18–24, 27–31]. The remaining ten studies examined non-alert patients unable to consciously report pain; this was most often in trauma, critical-care, or paediatric settings, where objective measurements were required because of altered consciousness or non-verbal status [14, 16, 17, 25, 26, 32–36].

### Criteria for compartment syndrome diagnosis and treatment

There was substantial heterogeneity in the minimum intracompartmental pressure thresholds used for diagnosis of compartment syndrome. Reported criteria varied depending on whether (1) the condition was acute or chronic and (2) absolute or differential (perfusion) pressure was used.

For CECS, the lowest diagnostic thresholds reported were 15 mmHg at rest, > 30 mmHg one minute post-exercise, and > 20 mmHg five minutes post-exercise [19, 29].

For ACS, absolute pressure thresholds were generally higher and often assessed relative to systemic blood pressure. Most studies adopted a fasciotomy threshold between 30 and 45 mmHg, consistent with current clinical consensus. Aedo-Martín et al. identified 30 mmHg as the most widely accepted absolute criterion [27]. In one study, the two patients who developed ACS and underwent emergency fasciotomy had intracompartmental pressures of 32 mmHg and 34 mmHg [16]. Another study defined an absolute threshold of > 40 mmHg for fasciotomy in revascularisation patients [32].

Conversely, several studies recommended decompression in equivocal cases when the differential pressure fell below 30 mmHg, while others proposed more stringent thresholds of < 20 mmHg for impending ischaemia [36] and 10 mmHg as a critical cutoff [34].

### Device characteristics and measurement protocols

The included studies employed a variety of near-infrared spectroscopy (NIRS)–based regional oximetry devices to assess their ability to detect or predict compartment syndrome. These systems aimed to determine whether reductions in tissue oxygen saturation could serve as a diagnostic surrogate for elevated intracompartmental pressures. Devices used included the INVOS oximeter (7 studies), InSpectra tissue spectrometer (6 studies), Run-Man NIRS spectrometer (3 studies), Biospectrometer-NB Oximeter (2 studies), Equanox oximeter (2 studies), Portamon (1 study) [15], CareGuideNIRS-pH (1 study) [20], and a custom device developed at K.J. Hospital (1 study) [16]. Considerable variation existed across studies in sensor placement, wavelength range, calibration methods, and output interpretation.

In most included studies, the lower leg was the primary region investigated. The NIRS probe was typically positioned over the mid-portion of the anterior compartment, directly overlying the tibialis anterior muscle [14, 16, 17, 19, 22–24, 29–31, 36]. In these studies, the intramuscular pressure (IMP) catheter was generally inserted into the same compartment to allow for direct comparison between local tissue oxygenation and ICP.

Other studies evaluated all four compartments (anterior, lateral, superficial posterior, and deep posterior) of the injured and contralateral control leg by placing sensors over the mid-portion of each muscle belly [14–16, 33, 34, 36]. NIRS-derived tissue oxygenation values were generally obtained from a depth of 2–3 cm beneath the skin surface, corresponding to the muscle region directly below the sensor [14, 16, 17, 23, 24, 29, 32–34, 36].

To establish a hyperaemic response or baseline perfusion level, several studies placed additional NIRS sensors on uninjured limbs or remote anatomical sites, including the volar forearm, deltoid, thigh, and instep of the foot [14, 19, 20, 27, 36]. However, some authors cautioned against using the deltoid muscle as a control site due to the high variability in readings observed across patients [14].

### Diagnostic accuracy and thresholds

The reported sensitivity and specificity of NIRS varied substantially across studies, influenced by the type of compartment syndrome investigated (acute vs. chronic), the diagnostic cutoff thresholds applied, and whether the study design was clinical or simulation-based (Table 2).

**Table 2:** Diagnostic accuracy of near-infrared spectroscopy (NIRS) for detecting compartment syndrome across included studies

References	Condition	Threshold/Metric	Sensitivity	Specificity	Key finding
Gentilello et al. [21]	Simulated ACS	StO_2_ vs. Perfusion Pressure (PP) (for detecting > 50% ischaemia)	85%	83%	NIRS had higher sensitivity than perfusion pressure for detecting ischemia defined by nerve conduction studies
van den Brand et al. [28]	CECS	StO_2_ ≤ 50% or ≤ 55% at peak exercise	78%	67%	Sensitivity was found to be comparable to Intracompartmental Pressure (ICP) measurements (77%)
van den Brand et al. [18]	CECS	≥ 35% decrease in StO_2_ from baseline	85%	67%	Sensitivity was found to be comparable to Intracompartmental Pressure (ICP) measurements reported in van den Brand et al. (2004)
Lee et al. [22]	Simulated ACS (ICP benchmark ≥ 30 mmHg	Differentiating normal from elevated IMP (> 30 mmHg)	65%	65%	NIRS was a slightly less accurate predictor compared to the co-tested PPLL technique (AUC 0.68 vs. 0.78)
Rennerfelt et al. [30]	CECS	T90 (time for StO_2_ to reach 90% of baseline) ≥ 30 s	38%	50%	Measures based on reoxygenation time also provided low sensitivity and specificity for diagnosis
Zhang et al. [31]	CACS	R90 ≥ 30 s (reoxygenation time)	60%	45%	The magnitude of intramuscular deoxygenation was found to be an unreliable measure for CACS

Summary of key studies assessing the diagnostic accuracy of NIRS in acute (ACS) and chronic exertional compartment syndrome (CECS). Reported parameters include diagnostic thresholds (e.g., StO_2_ or reoxygenation indices), sensitivity, specificity, and main findings. ACS, acute compartment syndrome; CECS, chronic exertional compartment syndrome; CACS, chronic anterior compartment syndrome; ICP, intracompartmental pressure; IMP, intramuscular pressure; PP, perfusion pressure; StO_2_, tissue oxygen saturation; T90, time to 90% StO_2_ recovery; R90, reoxygenation time; PPLL, partial pressure laser line; AUC, area under the curve

Near-infrared spectroscopy (NIRS) demonstrated a consistent and physiologically meaningful correlation with tissue ischemia across both simulated and clinical models of compartment syndrome. Unlike ICP monitoring, NIRS directly measures tissue oxygenation, providing an immediate indicator of cellular hypoxia [36]. In a study investigating simulated ACS, NIRS detected ischemia-induced neuromuscular blockade with greater sensitivity than ICP, confirming its capacity to identify critical reductions in tissue perfusion before irreversible damage occurred [21]. In clinical settings, NIRS values demonstrated a strong positive correlation with the compartmental perfusion, with reported correlation coefficients as high as r = 0.82 in patients with established ACS [34]; others have suggested that NIRS serves as a valuable adjunct to ICP monitoring in non-verbal patients, such as infants as young as one month old [25]. NIRS demonstrated similar sensitivity to ICP in detecting ACS [18, 28]. Similarly, controlled models of ACS revealed that StO_2_ remained stable until perfusion pressure declined below approximately 10 mm Hg, after which tissue oxygenation dropped precipitously [14, 23, 32].

Several studies also identified characteristic diagnostic patterns. Shuler et al. (2018) and Jagadeesan et al. (2022) reported that the loss of the normal hyperaemic gradient, defined as when the NIRS readings in the injured limb are typically ≥ 3% higher than the contralateral side, was a sensitive early indicator of evolving ACS [16, 36]. In all seven patients with confirmed ACS, at least one compartment demonstrated NIRS values ≥ 3% lower than the uninjured contralateral compartment, suggesting that relative inter-limb comparisons may provide a practical bedside surveillance tool [36].

Post-fasciotomy measurements further validated the physiological relevance of NIRS. In both Giannotti et al. (2000) and Arató et al. (2009), mean StO_2_ increased significantly following fasciotomy, confirming that NIRS accurately reflects restoration of tissue perfusion [14, 32].

### Studies reporting limited or no correlation between nirs and diagnostic criteria

Despite these promising findings, technical limitations were evident in acute trauma monitoring. Schmidt et al. (2018) demonstrated that continuous NIRS recording yielded substantially less complete and reliable data than ICP monitoring (median 31.6% vs. 87.4% valid data capture), with measurement artefacts attributed to hematoma, wound contamination, and sensor displacement [17].

Bariteau et al. found no significant relationship between StO_2_ and either absolute or differential compartment pressures in ACS, concluding that NIRS could not reliably substitute for pressure monitoring [33]. In Zhang et al., the magnitude of intramuscular deoxygenation during exercise did not differ significantly between chronic anterior compartment syndrome (CACS) patients and controls [31]. Similarly, Rennerfelt et al. observed minimal differences in peak-exercise StO_2_ and no correlation between perfusion pressure and reoxygenation times, suggesting limited diagnostic value [30]. Tønning et al. reported no association between StO_2_ changes and pain severity or exercise-induced leg-pain scores, with similar peak-exercise StO_2_ in CECS and control groups [15]. In Gustafsson et al., relative deoxygenation and recovery rates did not differ significantly between non-diabetic CECS patients and healthy participants [19]. Even in otherwise supportive studies, such as Giannotti et al., some confirmed ACS cases exhibited StO_2_ values comparable to the contralateral limb. This potential insensitivity led the authors to caution that NIRS alone may be insufficient to guide fasciotomy decisions [14].

### Quantitative synthesis of tissue oxygenation in compartment syndrome

**Fig. 2:**
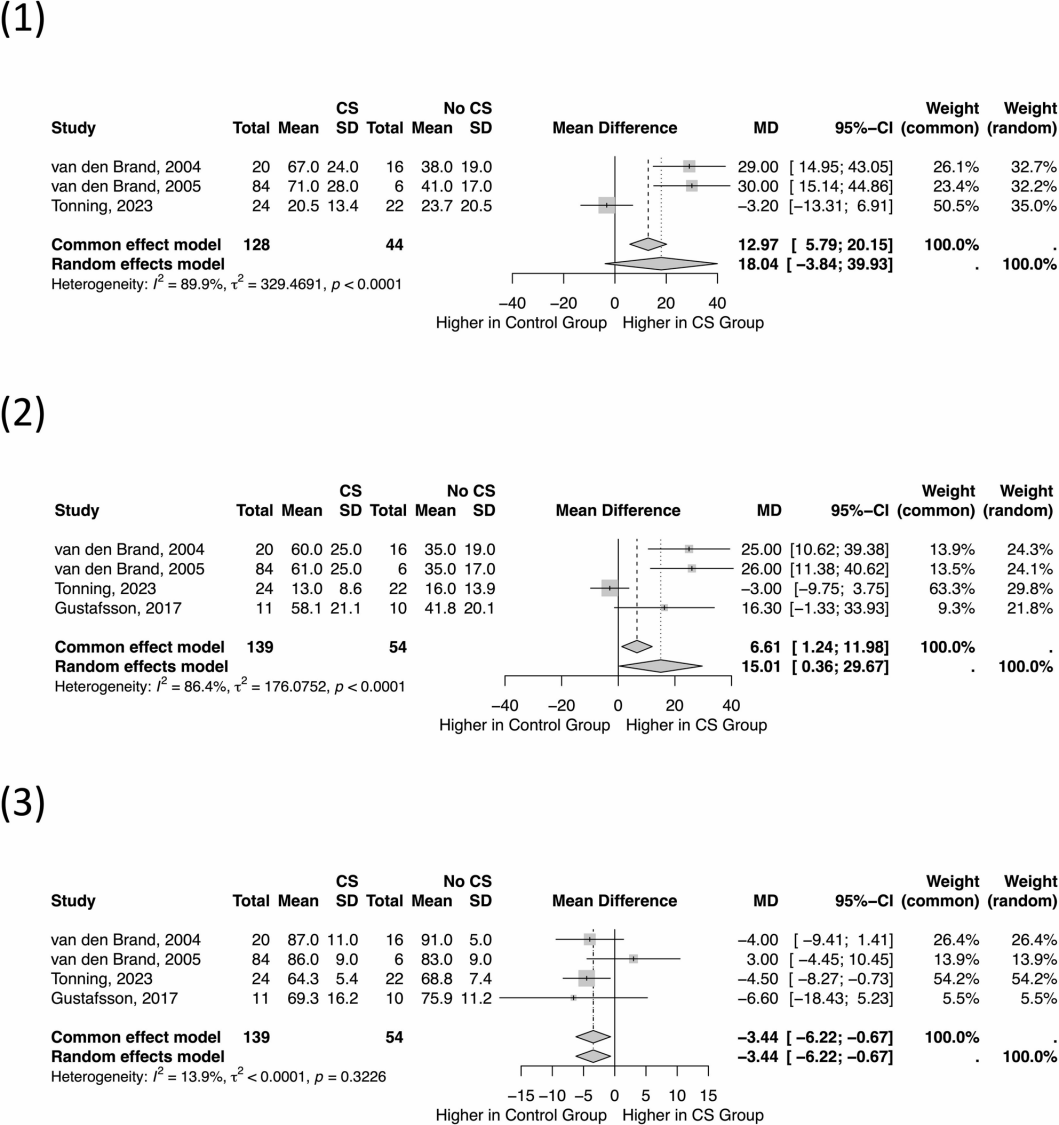
Forest plots showing pooled mean differences in (1) relative change in tissue oxygen saturation (StO_2_), (2) absolute change in StO_2_, and (3) baseline resting StO_2_ between compartment syndrome (CS) and control groups

A quantitative synthesis was performed using studies that reported NIRS-derived tissue oxygen saturation (StO_2_) in CECS cohorts with appropriate control data. Three key parameters were pooled across eligible studies: (1) relative change in StO_2_ between baseline and peak exercise, (2) absolute change in StO_2_ (percentage-point difference), and (3) baseline (resting) StO_2_ prior to exercise (Fig. 2).

#### *Relative change in StO*_2_* (percentage drop from baseline)*

CECS limbs exhibited a greater relative decline in StO_2_ during exercise compared with controls (pooled mean difference =  + 18.0%, 95% CI − 3.8 to 39.9; I^2^ = 90%, *p* < 0.01). Although the direction of effect was consistent, the confidence interval crossed zero, indicating non-significance at the 0.05 level. The high heterogeneity likely reflects methodological variation in NIRS device type, compartment selection, and exercise protocols. Nevertheless, the overall trend supports the hypothesis that CECS compartments experience exaggerated exercise-induced deoxygenation due to impaired microvascular perfusion.

#### *Absolute change in StO*_2_* (percentage-point difference)*

When expressed as the absolute numerical fall from baseline to lowest recorded StO_2_, CECS patients demonstrated significantly greater deoxygenation than controls (pooled mean difference =  + 15.0%, 95% CI 0.4 to 29.7; I^2^ = 86%, *p* < 0.01). This parameter captures the magnitude of ischemic decline without normalisation and may offer a more clinically comparable measure across devices. The consistent positive mean difference reinforces that NIRS effectively detects dynamic, exercise-induced hypoxia in affected compartments.

#### *Baseline StO*_2_* (resting tissue oxygenation)*

At rest, CECS patients demonstrated significantly lower StO_2_ compared with controls (pooled mean difference = − 3.4%, 95% CI − 6.2 to − 0.7; I^2^ = 14%). Although the absolute difference was small, it indicates a subtle but measurable perfusion deficit preceding exertion, consistent with previously reported elevated resting compartment pressures.

Taken together, the resting, relative, and absolute findings suggest that NIRS can detect physiologically meaningful perfusion differences at rest and during exercise, though baseline StO_2_ alone lacks diagnostic specificity.

## Discussion

The fundamental challenge in compartment syndrome diagnosis is that intracompartmental pressure (ICP), the current reference standard, measures a mechanical surrogate rather than the physiological endpoint of clinical concern: tissue ischemia. Our synthesis of 23 studies reveals that near-infrared spectroscopy (NIRS) directly addresses this limitation by quantifying tissue oxygenation, the actual determinant of cellular viability. Across both acute and chronic contexts, affected compartments demonstrated a consistent physiological signature: reduced tissue oxygen saturation (StO_2_) at baseline (mean difference − 3.4%, 95% CI − 6.2 to − 0.7) and exaggerated deoxygenation during ischemic stress (absolute change + 15.0%, 95% CI 0.4 to 29.7). To date, no synthesis has directly compared NIRS diagnostic performance across acute and chronic presentations or systematically evaluated demographic and technical influences on measurement accuracy. This convergence of findings establishes regional oximetry as a biologically valid method for detecting compartment-specific perfusion deficits, though substantial methodological heterogeneity limits definitive diagnostic thresholds.

In acute settings, NIRS reliably reflected ischemic changes preceding or coinciding with pressure thresholds traditionally associated with fasciotomy. Post-decompression increases in StO_2_ validated its responsiveness to restored perfusion. Collectively, the evidence suggests that NIRS offers a biologically relevant, non-invasive, and continuous means of detecting evolving ischemia, with particular value when clinical assessment or invasive pressure measurement are impractical.

The relationship between perfusion pressure and tissue oxygenation aligns with the pathophysiological continuum of compartment syndrome: once capillary perfusion pressure is exceeded by rising intracompartmental pressure, microvascular collapse and cellular hypoxia follow. Previous work demonstrated that NIRS could detect ischemia with greater sensitivity than ICP, a finding corroborated by high observed correlations between StO_2_ and perfusion gradient [21, 34]. Our pooled data extend these observations by showing that even small baseline reductions in StO_2_ precede exertional ischemia in chronic exertional compartment syndrome (CECS), consistent with elevated resting pressures and reduced capillary compliance [29].

Nevertheless, the diagnostic performance (used descriptively rather than as a formal metric of sensitivity and specificity) of NIRS across studies remains variable. Some series found weak or absent correlations with ICP [30, 33], likely reflecting heterogeneity in device, sensor positioning, exercise protocols, patient characteristics, and reference standards. Importantly, the directionality of effect was consistent: compartments affected by syndrome or experimental ischemia universally showed lower or more labile StO_2_ compared with controls. This reinforces the physiological validity of the measurement even when statistical thresholds were unmet.

The methodological diversity of NIRS studies may partly explain inconsistent sensitivity and specificity. Devices differ in wavelength range, algorithmic weighting of arterial versus venous blood, and sampling depth (1–3 cm). Because light penetration is proportional to emitter-detector spacing, subcutaneous fat thickness markedly influences accuracy. Studies suggest that even modest increases in adipose tissue layer thickness can significantly attenuate NIRS signal [37, 38], and a layer > 4 cm may serve as an exclusion criterion for NIRS [15]. The technique is thus most reliable in lean or muscular limbs, such as those of athletes or soldiers, which likely predominate in CECS cohorts; however, BMI was rarely reported, and none of the included studies statistically adjusted for subcutaneous fat thickness. Additionally, the limited penetration depth may fail to capture perfusion within deeper muscle compartments, particularly the deep posterior compartment of the leg.

In acute trauma, technical artefacts including hematoma, wound contamination, and sensor displacement contribute to data loss [17, 35]. Signal interference from external light and probe compression further compound variability. Continuous real-time quality control algorithms and improved fixation systems may mitigate these issues in future devices.

Only seven of twenty-three studies reported participant ethnicity. Melanin absorbs near-infrared wavelengths, reducing transmitted light and leading to underestimation of tissue oxygenation [39]. The studies varied in their racial demographics and did not stratify or adjust for pigmentation. For instance, some studies had majority Black patients [34, 36], whereas others included exclusively White patients [18, 28]. The absence of standardised reporting and calibration across skin tones represents a critical gap in the evidence base. Age, sex, and anatomical site may also modulate readings. Most data derive from young male populations with lower-leg involvement; few studies assessed thigh, forearm, or upper-limb compartments. Notably, Tobias and Hoernschemeyer demonstrated successful use of NIRS in a one-month-old infant with acute compartment syndrome (ACS), suggesting applicability across age ranges if device calibration accounts for tissue optical differences [25]. These demographic biases underscore the need for inclusive validation cohorts before NIRS can be universally endorsed, while ensuring equitable diagnostic accuracy.

Taken together, the current evidence supports a complementary rather than replacement role for NIRS in compartment syndrome diagnosis. Continuous monitoring provides a valuable early-warning system, particularly in sedated or obtunded patients where pain-based clinical signs are absent. Dynamic trends – such as a progressive decline in StO_2_ > 15% from baseline or a > 3% inter-limb difference – appear more informative than single absolute thresholds. Restoration of StO_2_ after fasciotomy confirms the physiological relevance of these changes. However, given inter-individual variability and technological limitations, NIRS should be interpreted alongside clinical findings and, where feasible, ICP measurement. Its greatest potential lies in guiding the timing of decompression, identifying evolving ischemia before irreversible necrosis, and reducing unnecessary fasciotomies in equivocal cases. Integration into trauma monitoring systems could provide continuous, non-invasive surveillance in critical care environments.

The available literature remains limited by small sample sizes, different patient characteristics, heterogeneous study designs, and inconsistent reference standards. Few studies correlate NIRS with definitive surgical findings or postoperative outcomes. Continuous acquisition reliability in acute trauma remains suboptimal, and device calibration across varying tissue compositions has not been standardised. Although this review followed PRISMA-ScR methodology to comprehensively map the field, heterogeneity precluded formal meta-analytic pooling beyond the CECS subgroup. Moreover, scoping reviews inherently prioritise breadth over quantitative precision. The pooled estimates presented here should therefore be interpreted as hypothesis-generating rather than definitive. Despite these limitations, physiological, experimental, and early clinical evidence converge in support of regional oximetry. This indicates that regional oximetry captures the essential pathophysiology of compartment syndrome. Structured validation in adequately powered, multi-centre prospective trials are therefore warranted. Such trials should: (1) enroll diverse cohorts stratified by ethnicity, sex, BMI, and age to establish population-specific calibration algorithms; (2) employ blinded outcome assessment with fasciotomy decision-making and functional outcomes (e.g., chronic pain, return to activity) as reference standards rather than ICP alone; (3) standardise device type, sensor placement anatomical landmarks, and measurement protocols to enable cross-site comparability; (4) establish minimum valid data capture thresholds as a prerequisite for per-protocol analysis; (5) prospectively define dynamic trend thresholds rather than relying on post-hoc optimal cutpoints that inflate apparent accuracy; (6) incorporate health economic analyses including costs, quality-adjusted life years, and rates of preventable fasciotomy or delayed diagnosis; and (7) develop machine-learning algorithms integrating NIRS trends with clinical variables, ICP when available, and patient-specific factors to generate individualized risk scores. Only through such rigorous, pragmatic validation can NIRS transition from promising investigational tool to evidence-based clinical standard. Importantly, fasciotomy decisions in such trials should still be influenced by established clinical criteria (e.g. reported pain out of proportion), with NIRS data collected concurrently for evaluation and not used to delay treatment.

## Conclusion

Regional oximetry using near-infrared spectroscopy offers a physiologically sound, non-invasive, and continuous method for detecting evolving ischemia in compartment syndrome. While not yet a replacement for intracompartmental pressure monitoring, it provides crucial complementary information, particularly in providing capacity for continuous, real-time monitoring in sedated or non-verbal patients or in paediatric settings, addressing key limitations of current diagnostic pathways. The present synthesis highlights robust evidence of perfusion deficits detectable by near-infrared spectroscopy (NIRS) in both acute and chronic syndromes, while emphasising the urgent need for standardisation across devices, populations, and protocols. With refinement and validation, NIRS has the potential to transform the diagnostic paradigm of compartment syndrome from static pressure measurement to dynamic physiological monitoring.

## Data Availability

Following PRISMA-ScR guidelines, PubMed, EMBASE, Cochrane Library, ClinicalTrials.gov, and WHO-ICTRP were searched to April 2025 for studies evaluating NIRS in acute (ACS) or chronic exertional (CECS) compartment syndrome. Data from these studies were included and used.

## Abbreviations

ACS: Acute compartment syndrome
AUC: Area under the curve
BMI: Body mass index
BP: Blood pressure
CACS: Chronic anterior compartment syndrome
CECS: Chronic exertional compartment syndrome
ICP: Intracompartmental pressure
IMP: Intramuscular pressure
NIRS: Near-infrared spectroscopy
PP: Perfusion pressure
PPLL: Partial pressure laser line
PRISMA: Preferred reporting items for systematic reviews and meta-analyses
PRISMA-ScR: Preferred reporting items for systematic reviews and meta-analyses extension for scoping reviews
R90: Reoxygenation time
SD: Standard deviation
StO_2_: Tissue oxygen saturation
T90: Time to 90% StO_2_ recovery
WHO-ICTRP: World Health Organization International Clinical Trials Registry Platform

## References

[1] Miciak M, Jurkiewicz K. Compartment syndrome—a complex and insidious medical problem. J Pre-Clin Clin Res. 2023. 10.26444/jpccr/163321.

[2] McMillan TE, Gardner WT, Schmidt AH, Johnstone AJ. Diagnosing acute compartment syndrome—Where have we got to? Int Orthop. 2019;43:2429–35. 10.1007/s00264-019-04386-y. 31468110 10.1007/s00264-019-04386-y PMC6848051

[3] Pechar J, Lyons MM. Acute compartment syndrome of the lower leg: a review. J Nurse Pract JNP. 2016;12:265–70. 10.1016/j.nurpra.2015.10.013. 27499719 10.1016/j.nurpra.2015.10.013 PMC4970751

[4] Lee C, O’Toole RV. Compartment syndrome in polytrauma patients. In: Mauffrey C, Hak DJ, Martin MP, editors. Compartment syndrome: a guide to diagnosis and management. Cham: Springer; 2019. p. 133–44.

[5] Buerba RA, Fretes NF, Devana SK, Beck JJ. Chronic exertional compartment syndrome: current management strategies. Open Access J Sports Med. 2019;10:71–9. 10.2147/OAJSM.S168368. 31213933 10.2147/OAJSM.S168368 PMC6537460

[6] British Orthopaedic Association. Diagnosis and management of compartment syndrome of the extremities (BOAST). London: British Orthopaedic Association; 2025.

[7] Vogels S, de Vries D, Bakker EWP, et al. Measuring intracompartmental pressures in the lower leg: assessing the use of unilateral measurements in patients with bilateral symptoms. JB JS Open Access. 2022. 10.2106/JBJS.OA.22.00041. 36447496 10.2106/JBJS.OA.22.00041 PMC9699657

[8] Scheeren TWL, Schober P, Schwarte LA. Monitoring tissue oxygenation by near infrared spectroscopy (NIRS): background and current applications. J Clin Monit Comput. 2012;26:279–87. 10.1007/s10877-012-9348-y. 22467064 10.1007/s10877-012-9348-y PMC3391360

[9] Cole AL, Smith EK, Austin AV, et al. Near-infrared spectroscopy monitoring for compartment syndrome. Tech Orthop. 2012. 10.1097/bto.0b013e31824881f6.

[10] Tricco AC, Lillie E, Zarin W, et al. PRISMA extension for scoping reviews (PRISMA-ScR): checklist and explanation. Ann Intern Med. 2018;169:467–73. 10.7326/M18-0850. 30178033 10.7326/M18-0850

[11] Cochran WG. Some methods for strengthening the common χ 2 tests. Biometrics. 1954;10:417. 10.2307/3001616.

[12] Higgins JPT, Thompson SG. Quantifying heterogeneity in a meta-analysis. Stat Med. 2002;21:1539–58. 10.1002/sim.1186. 12111919 10.1002/sim.1186

[13] Deeks JJ, Higgins JP, Altman DG, on behalf of the Cochrane Statistical Methods Group. Analysing data and undertaking meta-analyses. In: Cochrane Handbook for Systematic Reviews of Interventions. 2019. pp. 241–284.

[14] Giannotti G, Cohn SM, Brown M, et al. Utility of near-infrared spectroscopy in the diagnosis of lower extremity compartment syndrome. J Trauma Injury Infect Crit Care. 2000. 10.1097/00005373-200003000-00005. 10.1097/00005373-200003000-00005 10744275

[15] Tønning LU, Mygind-Klavsen B, Kjeldsen T, et al. Muscle strength, oxygen saturation and physical activity in patients with chronic exertional compartment syndrome compared to asymptomatic controls. Int J Sports Phys Ther. 2023. 10.26603/001c.71357. 37020455 10.26603/001c.71357 PMC10069367

[16] Jagadeesan K, John AM, Ramachandran V, John PS. Near infrared spectroscopy in monitoring compartment pressure. J Int Med Sci Acad. 2022;35:209–12.

[17] Schmidt AH, Bosse MJ, Obremskey WT, et al. Continuous near-infrared spectroscopy demonstrates limitations in monitoring the development of acute compartment syndrome in patients with leg injuries. J Bone Joint Surg. 2018;100:1645–52. 10.2106/JBJS.17.01495. 30277994 10.2106/JBJS.17.01495

[18] Van Den Brand JGH, Nelson T, Verleisdonk EJMM, Van Der Werken C. The diagnostic value of intracompartmental pressure measurement, magnetic resonance imaging, and near-infrared spectroscopy in chronic exertional compartment syndrome: a prospective study in 50 patients. Am J Sports Med. 2005;33:699–704. 10.1177/0363546504270565. 15722275 10.1177/0363546504270565

[19] Gustafsson P, Crenshaw AG, Edmundsson D, et al. Muscle oxygenation in Type 1 diabetic and non-diabetic patients with and without chronic compartment syndrome. PLoS ONE. 2017;12:e0186790. 10.1371/journal.pone.0186790. 29059243 10.1371/journal.pone.0186790 PMC5653333

[20] Challa ST, Hargens AR, Uzosike A, Macias BR. Muscle microvascular blood flow, oxygenation, pH, and perfusion pressure decrease in simulated acute compartment syndrome. J Bone Jt Surg. 2017;99:1453–9. 10.2106/JBJS.16.01191. 10.2106/JBJS.16.01191 PMC5685422 28872527

[21] Gentilello LM, Sanzone A, Wang L, et al. Near-infrared spectroscopy versus compartment pressure for the diagnosis of lower extremity compartmental syndrome using electromyography-determined measurements of neuromuscular function. J Trauma Injury Infect Crit Care. 2001. 10.1097/00005373-200107000-00001. 10.1097/00005373-200107000-00001 11468459

[22] Lee SH, Padilla M, Lynch JE, Hargens AR. Noninvasive measurements of pressure for detecting compartment syndromes. J Orthop Rheumatol. 2013;1:5. 25328908 PMC4197938

[23] Reisman WM, Shuler MS, Kinsey TL, et al. Relationship between near infrared spectroscopy and intra-compartmental pressures. J Emerg Med. 2013;44:292–8. 10.1016/j.jemermed.2012.06.018. 22921857 10.1016/j.jemermed.2012.06.018

[24] Briet GA, Gross JH, WATENPAUGH DE, et al. Near-infrared spectroscopy for monitoring of tissue oxygenation of exercising skeletal muscle in a chronic compartment syndrome model. JBJS. 1997;79:838–43. 10.2106/00004623-199706000-00006 9199380

[25] Tobias JD, Hoernschemeyer DG. Near-infrared spectroscopy identifies compartment syndrome in an infant. J Pediatr Orthop. 2007. 10.1097/BPO.0b013e3180326591. 17414016 10.1097/BPO.0b013e3180326591

[26] de Sanchez Toledo J, Chrysostomou C, Wearden PD. Acute compartment syndrome in a patient on extracorporeal support: utility of near-infrared spectroscopy. J Cardiothorac Vasc Anesth. 2011;25:836–7. 10.1053/j.jvca.2010.06.023. 20832334 10.1053/j.jvca.2010.06.023

[27] Aedo-Martín D, Navarro-Suay R, García-Cañas R, et al. Use of oxygen tissue monitoring in patients with compartment syndrome: two clinical cases and literature review. Mil Med. 2019;184:e475–9. 10.1093/milmed/usy270. 30371908 10.1093/milmed/usy270

[28] Van Den Brand JGH, Verleisdonk EJMM, Van Der Werken C. Near infrared spectroscopy in the diagnosis of chronic exertional compartment syndrome. Am J Sports Med. 2004;32:452–6. 10.1177/0363546503261733. 14977673 10.1177/0363546503261733

[29] Mohler LR, Styf JR, Pedowitz RA, et al. Intramuscular deoxygenation during exercise in patients who have chronic anterior compartment syndrome of the leg*. J Bone Joint Surg Am. 1997. 10.2106/00004623-199706000-00007. 9199381 10.2106/00004623-199706000-00007

[30] Rennerfelt K, Zhang Q, Karlsson J, Styf J. Changes in muscle oxygen saturation have low sensitivity in diagnosing chronic anterior compartment syndrome of the leg. J Bone Joint Surg Am. 2016. 10.2106/JBJS.N.01280. 26738904 10.2106/JBJS.N.01280

[31] Zhang Q, Rennerfelt K, Styf J. The magnitude of intramuscular deoxygenation during exercise is an unreliable measure to diagnose the cause of leg pain. Scand J Med Sci Sports. 2012;22:690–4. 10.1111/j.1600-0838.2011.01392.x. 22092660 10.1111/j.1600-0838.2011.01392.x

[32] Arató E, Kürthy M, Sínay L, et al. Pathology and diagnostic options of lower limb compartment syndrome. Clin Hemorheol Microcirc. 2009;41:1–8. 10.3233/CH-2009-1145. 19136736 10.3233/CH-2009-1145

[33] Bariteau JT, Beutel BG, Kamal R, et al. The use of near-infrared spectrometry for the diagnosis of lower-extremity compartment syndrome. Orthopedics. 2011;34:178. 10.3928/01477447-20110124-12. 21410124 10.3928/01477447-20110124-12

[34] Shuler MS, Reisman WM, Kinsey TL, et al. Correlation between muscle oxygenation and compartment pressures in acute compartment syndrome of the leg. J Bone Jt Surg-Am. 2010;92:863–70. 10.2106/JBJS.I.00816. 10.2106/JBJS.I.00816 20360509

[35] Shuler MS, Reisman WM, Cole AL, et al. Near-infrared spectroscopy in acute compartment syndrome: case report. Injury. 2011;42:1506–8. 10.1016/j.injury.2011.03.022. 21489528 10.1016/j.injury.2011.03.022

[36] Shuler MS, Roskosky M, Kinsey T, et al. Continual near-infrared spectroscopy monitoring in the injured lower limb and acute compartment syndrome: an FDA-IDE trial. Bone Joint J. 2018;100-B:787–97. 10.1302/0301-620X.100B6.BJJ-2017-0736.R3. 29855235 10.1302/0301-620X.100B6.BJJ-2017-0736.R3

[37] Niemeijer VM, Jansen JP, van Dijk T, et al. The influence of adipose tissue on spatially resolved near-infrared spectroscopy derived skeletal muscle oxygenation: the extent of the problem. Physiol Meas. 2017;38:539. 10.1088/1361-6579/aa5dd5. 28151429 10.1088/1361-6579/aa5dd5

[38] van Beekvelt MC, Borghuis MS, van Engelen BG, et al. (2001) Adipose tissue thickness affects in vivo quantitative near-IR spectroscopy in human skeletal muscle. Clin Sci Lond Engl. 1979;101:21–8. 10.1042/cs20000247. 10.1042/cs20000247 11410110

[39] Roy S, Wu J, Cao J, et al. Exploring the impact and influence of melanin on frequency-domain near-infrared spectroscopy measurements. J Biomed Opt. 2024;29:S33310. 10.1117/1.JBO.29.S3.S33310. 39323492 10.1117/1.JBO.29.S3.S33310 PMC11423252

